# Books Reviewed

**Published:** 1864-12

**Authors:** 


### BOOKS REVIEWED.

*Outlines of Surgical Diagnosis, by* GEORGE H. B. MACLEOD, M. D., F. R. C. S. E.; *El. Fac. Phys. and Surg. Glasgow; Lecturer on Surgery, Anderson's University; Surgeon to the Glasgow Royal Infirmary, and the Lock Hospital; late Senior Surgeon, Civil Hospital Smyrna, and General Hosqital in Camp before Sebastopol; Mem, Cor. De La Soc. de Chir. de Paris; and author of "Notes on the Surgery of the War in the Crimea." First American edition, reprinted from advance sheets. New York:* BAILLIERE BROTHERS, 510 *Broadway*, 1864.

The art of recognizing disease and of distinguishing one from another, is of such necessity as to be indispensable in the successful practice of surgery. An extensive and accurate knowledge of disease, and the various symptoms by which disease is manifested, and an acquaintance with the pathological conditions upon which these signs depend, is necessary in order to discriminate the precise nature of most surgical affections. If there is failure in this, the practice must become empirical. In the medical treatment of disease, symptoms are treated with some relief even when the exact pathology is undetermined, while in surgery it is often absolutely necessary to arrive at positive knowledge as to the nature of the malady before surgical or operative measures can with safety be adopted. Many surgical diseases terminate by natural tendency in health and require no interference; these it is only necessary to clearly discern; while others are so certainly fatal, by a tendency which we have no power to control, that we are only to attempt the relief of the more distressing effects, still correct diagnosis is such cases, even, is essential.

The power of discriminating with accuracy one disease from another, is that which elevates and distinguishes surgeons, and gives to some an almost intuitive knowledge of disease; an element of the greatest importance in the character of a practitioner.

While this power is to some extent a natural gift which all do not possess in equal degree, still education is capable of developing and improving it so that by attention and patient study we may all attain reasonable accuracy and be able to draw logical and just conclusions.

This work, on the outlines of surgical diagnosis, is written in terse and direct style, and each subject is treated fully and plainly. The symptoms of disease are given with clearness; diseases which may be confounded, are arranged and classified with the distinguishing features of each—diseases placed side by side are compared with each other, that the differences may appear the more obvious. The various methods of diagnosis—the means we possess of determining the nature of disease, are presented with great accuracy and force. The arrangement of the work is excellent, and a wide range of topics are brought within the limits of a volume of five hundred pages; this is done by using as many, and no more words, than are necessary to convey the author's idea. This book is an important addition to a surgeon's library; it has excellencies which we have not time to mention. If we had space, we should speak at length of some of the chapters, especially those on fractures and dislocations. In the small space devoted to consideration of these accidents is included just what practitioners desire most of all to know. As illustration of the condensed form of the work we quote one paragraph which is representative of the whole book: "In all dislocations of the humerus we observe—(a) Flattening of the shoulder. (b) Projection of the acromion process. (c) Hollow immediately below the acromion, in the position of the glenoid cavity. (d) The head of the bone is in abnormal position. (e) The length of the upper arm altered. (f) The axis of the humerus changed. (g) The motion (voluntary and communicated) of the articulation impaired. (h) The elbow in an abnormal position. (i) Pain, especially on movement."

It will thus be seen that the art of diagnosis is communicated in small space, while the beauty of direct composition is given in forcible examples. This is a new book, which we earnestly advise physicians to read.

---

*Therapeutics and Materia Medica; a Systematic Treatise on the Action, and Uses of Medical Agents, including their Description and History, by* ALFRED STILLE, M. D., *Professor of the Theory and Practice of Medicine in the University of Pennsylvania; Physician to St. Joseph's Hospital; Fellow of the College of Physicians, and member of the Pathological Society of Philadelphia; Member of the Societe Medicale D'Observation, of Paris; Honorary Member of the Medical Society of the State of Rhode Island, of the State of New York, etc. Second edition, revised and enlarged, in two volumes. Philadelphia:* BLANCHARD & LEA, 1864.

Stille's Therapeutics and Materia Medica has been so extensively used as a standard authority since the appearance of the first edition in 1860, that there seems to be no occasion to speak of its character and merits. It has become the text-book in most of our medical schools, and is everywhere regarded as the most complete and comprehensive work of the kind in the English language.

The second edition has been improved by a "thorough revision, and by incorporating into it whatever appeared to constitute a real advance in therapeutical knowledge." "The nomenclature of the materia medica and the formulæ for officinal preparations have been made to conform to the recent edition of the pharmacopœia, while a few medicines of minor importance contained in the first issue have been omitted. On the other hand, several new medicines have been introduced, and to almost every article important additions have been made. These in the aggregate, amount to over one hundred pages."

It is within the scope of this work to fully consider medicines in their physicial, chemical and physiological properties, and this has been done with a completeness which leaves nothing to be added.

The student who gains his views of the value of medicine from reading our standard works will be likely to hold to opinions which experience will greatly change. A great many articles are introduced into all systematic works upon materia medica more from a desire to make them comprehen. sive and full—a dictionary of materia medica, than on account of any supposed value in the articles themselves. All that medicine can do in the relief or cure of disease, can be done with a very limited number of medicines, and when we see a volume of such proportions as the one before us devoted to the therapeutical properties of drugs, we cannot repress the feel, ing that in many cases their uses and value are magnified "a great many diameters." These facts however do not detract in the least from such a work as the one before us. Medicines should be thoroughly studied that it may be fully known how little many of them are worth. There is no more reliable source, from which such knowledge may be obtained than *Stillé's Therapeutics and Materia Medica.*

---

*A System of Surgery; Pathological, Diagnostic, Therapeutic and Operative, by* Samuel D. Gross, M. D., *Professor of Surgery in the Jefferson College of Philadelphia; Surgeon to the Philadelphia Hospital; Member of the Imperial Royal Medical College of Vienna, etc., etc., illustrated by over thirteen hundred engravings. Third edition, much enlarged and carefully revised. In two volumes. Philadelphia:* Blanchard & Lea, 1864.

The third opportunity is now offered during our editorial life to review, or rather to endorse and recommend this great American work on Surgery. Upon this last edition a great amount of labor has been expended, though to all others except the author the work was regarded in its previous editions as so full and complete as to be hardly capable of improvement. Every chapter has been revised; the text augmented by nearly two hundred pages, and a considerable number of wood-cuts have been introduced. Many portions have been entirely re-written; and the additions made to the text, are principally of a practical character.

This comprehensive treatise upon Surgery has undergone revisions and enlargements, keeping pace with the progress of the art and science of surgery, so that whoever is in possession of this work, may consult its pages upon any topic embraced within the scope of its department and rest satisfied that its teaching is fully up to the present standard of surgical knowledge. It is also so comprehensive that it may truthfully be said to embrace all that is actually known, that is really of any value in the diagnosis and treatment of surgical diseases and accidents.

Wherever illustration will add clearness to the subject, or make better or more lasting impression, it is not wanting; in this respect the work is eminently superior. The publishers have also done their part in the most perfect manner; they have furnished clear type, white paper, and leather binding, which in these days of high prices, is especially liberal. The profession should appreciate the favor of being furnished these valuable works in so substantial form.

We do not attempt anything like review of such a work; our pages will not admit of it. Our readers are mostly familiar with its merits as a standard work, and we only desire to announce the appearance of the third edition, with its additions and improvements.

---

*Lectures on Venereal Diseases, by* WM. A. HAMMOND, M. D. *Philadelphia:* J. B. LIPPINCOTT & Co., 1864.

Some of these lectures were delivered at the Baltimore Infirmary in 1861, and published in the *American Medical Times;* the remainder were prepared but not given or published. They are now brought up to the present day, and made to embody the recent essential facts connected with venereal diseases.

In the treatment of soft chancre he says: "Now there are two circumstances which should pre-eminently influence us in the treatment of the simple chancre. 1st—You must not forget that it is altogether a local disease. 2d—That it is liable to extensive ulceration and phagedena. The former fact does away with any necessity for the exhibition of mercury, and the latter renders such a course not only improper, but highly dangerous. At the same time it is desirable to destroy as soon as possible the specific character of the chancre, and to convert it into a simple non-virulent ulcer."

Also upon the treatment of the infecting chancre we quote one paragraph: "In order therefore to prevent the syphilitic infection of the system from the virus of an indurated chancre—and no other can produce this result—two measures are necessary: First to destroy the chancre with some powerful caustic, as the carbo-sulphuric acid paste, monohydrated nitric acid or bromine; and second, to bring the system under the influence of mercury. The first should be done within six days after the appearance of the chancre, and the second commenced at the same time and carried out as rapidly as possible consistent with thoroughness. Even with all diligence you will sometimes fail in your endeavors, but you will often succeed. I have never, however seen constitutional infection prevented unless the chancre was destroyed within six days after its commencement, nor unless the system was well under the influence of mercury before the fifteenth day."

This is the old doctrine of Ricord which we supposed was no longer believed, and never would have been entertained had a distinction been made between chancre and chancroid. That a chancre is never a mere local legion is proved by the fact, as shown by repeated experiments, that its destruction within a few days or even hours after its appearance does not prevent constitutional infection. That mercury is necessary in the treatment of chancre is by no means probable; though in some cases it seems to have the effect the earlier to remove the induration and possibly the sooner to heal the ulcer. That the system should be brought as speedily as possible under its influence, appears by no means established, indeed it would seem almost certain that such medication is not only unnecessary but positively injurious. If the mercury is withheld until the supervention of secondary symptoms, the disease appears less virulent and more readily yields to the influence of that drug, which in this stage, has unmistakable control. The book has many merits and will prove a valuable, practical guide to the practitioner. It cannot be expected as yet that the profession will fully agree upon all the important questions involved in this subject. They are being determined by repeated experiment and careful observation; and every honest worker is thrice welcome to the results of his labor.

---

*Proceedings of the Nineteenth Annual Meeting of the Ohio State Medical Society, June 21st, 22d,* 1864.

We are indebted to Dr. E. B. Stevens, Secretary of the Ohio State Medical Society, for a copy of the proceedings of the meeting held at White Sulphur Springs June 21st, 22d, 1864.

It contains, in addition to the report of transactions, Valedictory Address by W. T. Kincaid, M. D., retiring President; Report on Diseases of the Eye, by A. Metz, M. D.; Report on New Remedies, by Edward B. Stevens, M. D., Editor of *Cincinnati Lancet and Observer;* Report on Asthma by Committee, by Thaddeus A. Reamy, M. D., and some notes of cases, lists of members, &c., &c. The papers presented to the society are all well written and valuable contributions; the one on New Remedies, by Dr. Stevens, has appeared in full in the pages of our journal, and is well worthy attentive perusal. Those who are fond of trying the last new remedy, will do well to consider it attentively; possibly it will modify their views and change the direction in which their ambition so strongly tends,

*Annals of the Medical Society of the County of Albany,* 1806–1851, *with Biographical Sketches of Deceased Members. By* SYLVESTER D. WILLARD, M. D. *Albany:* J. MUNSELL, 78 *State street,* 1864.

It is unbecoming a learned profession in this day "of letters," that its early history and the names and character of its members should remain unwritten. Dr. Willard has done for Albany County Medical Society what it is greatly to be desired some one should do for the other County Societies in the State. This Society has existed for more than half a century, and those who were its founders are for the most part numbered among its deceased members. The volume contains the records of the meetings of the Society from its beginning, with biographical sketches of its deceased members; it is embellished with lithographic likenesses of Drs. James McNaughton, John V. P. Quackenbush, Howard Townsend, and James Wade, former Presidents of the Society. It forms a volume of great local and historical interest, in which the author has set an example which some one should follow in every county in the State.

